# Development and implementation of the Clinical Tooth Shade Differentiation Course – an evaluation over 3 years

**DOI:** 10.3205/zma001001

**Published:** 2016-02-15

**Authors:** Constanze Olms, Rainer Haak, Holger A. Jakstat

**Affiliations:** 1 University of Leipzig, Department of Prosthodontics and Material Science, Germany; 2 University of Leipzig, Department of Cariology, Endodontology and Periodontology, Germany

**Keywords:** dental education, evaluation report, tooth shade differentiation, Toothguide Training Box

## Abstract

**Objective: **Tooth shade differentiation concerns the identification and classification of tooth shades. The objective of this project was to implement the Clinical Tooth Shade Differentiation Course in the preclinical stage of studies and to evaluate the students' perspective over a period of 3 years.

**Methodology: **The course is planned for a duration of 10 weeks with two 45-minute sessions per semester week. The entire attendance time was 10:15 h. 2 lectures of 90 minutes each, 2 seminars of 60 min each and 2 teaching units with the phantom head and role playing took place. In addition to the various parameters of tooth shade, changes in tooth shade and the basics of dental esthetics, clinical procedures for manual and digital tooth shade determination were explained and practiced. 96% (69 of 72) of the students participated in the first evaluation in 2012/2013 (T_1_), and 68% of these were women. In the following year, 2013/2014 (T_2_), 92% (45 of 48 students) took part; 62% of these were women and 38% men. The 2014/2015 evaluation (T_3_) comprised 94% (45 of 48 students). Of these, 67% were women.

**Results: **In the evaluation, the students gave the course a positive grade. The questions in "General/Organization" were given a mean (M) of 1.5 (SD=0.7) in T_1_ and T_2_ , and 1.2 (SD=0.3) in T_3_. The "Overall Assessment" yielded M_T1_=1.6 (SD=0.6), M_T2_=1.5 (SD=0.5) and M_T3_=1.1 (SD=0.3). In T_1_ and T_2_, the item "The instructor actively involved the students in the course" was given a mean of 2.1 (SD=0.9), and in T_3_ a mean of 1.2 (SD=0.5).

**Conclusions: **The course presented here conceptually shows how practical dental skills can be taught in a theoretical and clinical context. Educational objectives from the role of a dental expert were taken from the national competence-based catalog of educational objectives for dentistry and can also be supplemented. The objectives can be transferred to other dental faculties.

## Zusammenfassung

**Zielsetzung: **Die Zahnfarbdifferenzierung beschäftigt sich mit dem Erkennen und Einordnen von Zahnfarben. Das Ziel war es, die Lehrveranstaltung klinische Zahnfarbdifferenzierung in den vorklinischen Studienabschnitt zu implementieren und die Sicht der Studierenden über einen Zeitraum von 3 Jahren zu evaluieren.

**Methodik: **Die Lehrveranstaltung ist in einem Umfang über 10 Wochen mit zweimal 45 Minuten pro Semesterwoche angelegt. Die Gesamtpräsenszeit umfasste 10:15h. Es fanden 2 Vorlesungen à 90 min, 2 Seminare à 60 min und 2 Unterrichtseinheiten am Phantomkopf und im Rollenspiel statt. Neben den verschiedenen Parametern der Zahnfarbe sowie den Veränderungen der Zahnfarbe und den Grundlagen der zahnbezogenen Ästhetik, wurde die klinisch praktische Vorgehensweise in der manuellen und digitalen Zahnfarbbestimmung erläutert und trainiert. An der ersten Evaluation 2012/2013 (T_1_) nahmen 96% (69 von 72) der Studierenden teil, davon waren 68% weiblich. Im darauffolgenden Jahr 2013/2014 (T_2_) nahmen 92% (45 von 48 Studierenden) teil, davon waren 62% weiblich und 38% männlich. Die Evaluation 2014/2015 (T_3_) schloss 94% (45 von 48 Studierenden) ein. Davon 67% weiblichen Geschlechts.

**Ergebnisse:** Die Evaluierung der Studierenden zeigte eine positive Benotung dieser Lehrveranstaltung. Die Fragen zu „Allgemeines /Organisation“ wurde zu T_1_ und T_2_ im Mittelwert (M) mit der Schulnote 1,5 (SD=0,7) bewertet und zum Zeitpunkt T_3_ mit 1,2 (SD=0,3). Die „Gesamtbewertung“ ergab zu M_T1_=1,6 (SD=0,6), zu M_T2_=1,5 (SD=0,5) und zu M_T3_=1,1 (SD=0,3). Zu T_1_ und T_2_ wurde das Item „Der Dozent bezog die Studierenden aktiv in die Lehrveranstaltung ein“ noch mittelwertig mit 2,1 (SD=0,9) benotet, zu T_3_ im Mittel mit der Note 1,2 (SD=0,5).

**Schlussfolgerungen: **Die hier vorgestellte Lehrveranstaltung zeigt konzeptionell wie die zahnärztlichen praktischen Fertigkeiten zum theoretischen und klinischen Kontext vermittelt werden können. Aus dem nationalen kompetenzbasierten Lernzielkatalog Zahnmedizin wurden Lernziele aus der Rolle des Zahnmedizinischen Experten bereits übernommen bzw. können weiter ergänzt werden. Eine Übertragbarkeit auf andere zahnmedizinische Fakultäten ist dadurch gegeben.

## Introduction

In dentistry, esthetics and tooth shade are closely related. It is very important for patients to have a restoration that matches their natural teeth. Selecting the tooth shade for a patient is therefore an important step in treatment. Studies have shown that 65% of prosthetic restorations cannot be placed at the first appointment because the tooth shade has to be adjusted [1]. This is associated with unplanned treatment appointments for the patient and the dentist as well as additional costs for correcting shade changes at the dental laboratory [2]. 

For clinical tooth shade determination, dentists have visual and electronic aids at their disposal that allow communication between the dental office and the dental laboratory. 

A study that surveyed European students of dental medicine in 2011 showed that visual shade determination using the Vitapan Classical (17–67%) shade guide (Vita Zahnfabrik, Bad Säckingen, Germany) was the most commonly taught shade-taking procedure, followed by the Vita 3D-Master shade guide (0–47%) (Vita Zahnfabrik, Bad Säckingen, Germany). Only a small percentage of students (2–47%) had been introduced to a digital shade-taking procedure [3]. 

Various training systems have been developed for differentiating between tooth shades. In addition to exercises exclusively in shade training [4], [5], practice programs are also available that are designed for a particular shade-taking system, such as the Toothguide Training Box (TTB) curriculum developed at the University of Leipzig in Germany. The TTB curriculum has been part of the preclinical education in Leipzig since 2004 [6] and was specially designed for the procedure with the Vita 3D-Master. It consists of a Toothguide Trainer (TT) software program and the TTB. The TT displays a virtual VITA 3D-Master guide on the monitor. The user learns how to use the VITA 3D-Master in three steps (1. Lightness, 2. Chroma, 3. Hue). This is followed by training with the TTB using a real integrated shade guide under standardized lighting conditions. The exercises correspond to the procedure with the TT, with the exception that ceramic shade samples are used under daylight conditions. Examinations carried out independently of one another on users with normal vision showed that this training had a positive effect on the ability to differentiate between tooth shades [6], [7], [8], [9], [10], [11]. However, this training effect with TT and TTB decreases again after only 6 months [12]. 

The TTB curriculum trains the procedure of the VITA 3D-Master, but it is not oriented to a clinical situation with a patient. 

At the University of Leipzig, a needs analysis yielded that the teaching contents of tooth shade determination were not optimally coordinated between the preclinical and clinical stage. In preclinical training, tooth shade differentiation ability was taught in the framework of the TTB curriculum in the 2^nd^ or 3^rd^ semester, based on the Vita 3D-Master. However, there was no link to a clinical context. In clinical training, the Vitapan Classical shade guide was used for the visual shade-taking procedure. This means that the modern shade training system in preclinical training did not match the conventional shade determination in the clinical courses. Digital tooth shade determination was not part of the preclinical or clinical training either. 

In order to give students of dental medicine in Leipzig a uniform concept of shade differentiation, it was necessary to offer the corresponding educational objectives in a course before the start of the first clinical semester.

The objective of the current project was to describe the implementation of the Clinical Tooth Shade Differentiation Course and to investigate the perspective of the students over a period of three years.

## Project description

To implement the Clinical Tooth Shade Differentiation Course, the phantom course of Prosthodontics II in the 5^th^ preclinical semester was selected. This is held each year in the winter semester. The Tooth Shade Determination Course was included in the curriculum in Winter Semester 2012/13 and integrated in the existing phantom course II. It had a duration of 10:15 h, spread out over 10 semester weeks with a time frame of two 45 minute sessions. 

The methodical-didactic implementation is shown in table 1 (Tab. 1). The individual sessions alternated between lectures, seminars and practical exercises. The sequence of the lectures and seminars was oriented according to the sandwich principle [13], [14], [15], [16]. With the sandwich principle, keynote presentations alternated with individual and group work. 

The TT was offered to the students as an e-learning program. This allowed them to practice the procedure with the Vita 3D-Master on their own computers. Training with the TTBs then took place. This served as preparation for tooth shade differentiation with the phantom head. Practical skills for manual and digital shade-taking were presented and practiced in small groups (4-6 students) according to the Peyton method [17]. In a simulated clinical setting, tooth shades were selected using a phantom head equipped with extracted human teeth. The manual shade-taking procedure was performed with the Vita 3D-Master shade guide. A shade sample was assigned to the tooth to be determined in three consecutive steps,

1. Lightness,
2. Chroma, and
3. Hue.

For the digital shade-taking procedure, the measurement was done on the tooth surface with a spectrophotometer (Vita Easyshade, Vita Zahnfabrik, Bad Säckingen, Germany). Afterwards, the students determined each other's tooth shades in role playing, first with the Vita 3D-Master shade guide and then with the Vita Easyshade digital tooth shade-taking device on a dental treatment unit under the supervision of a dentist.

The following educational objectives were defined in the concept of this course. The educational objectives were:

1. To name the physical and physiological aspects of color theory.
2. To know, compare and use tooth shade differentiation in dentistry.
3. To explain the different parameters of tooth shade, as well as the physiological and other changes.
4. To apply the basic rules of dental esthetics.
5. To perform the necessary steps for visual tooth shade determination with the Vita 3D-Master in a clinical setting.
6. To use the intraoral spectrophotometer Vita Easyshade in a clinical setting.
7. To evaluate, describe and communicate the selected tooth shade in respect to esthetics.

Educational objectives 3 and 4 were taken from the chapter on dental experts in the national competence-based catalog of educational objectives for dentistry http://www.nklz.de]. 

At the end of the course, an evaluation was carried out. The evaluation period was three years. In Winter Semester 2012/2013 (T_1_), 72 students were enrolled and eligible to take part in the phantom course II, in Winter Semester 2013/2014 (T_2_) there were 60 students. And in Winter Semester 2014/2015 (T_3_) there were 48 students. 

Standardized questionnaires of the Leipzig Faculty of Medicine for evaluating new courses were used. For this purpose, the EvaSys program version 6.0 (Electric Paper Evaluationssysteme GmbH, Lüneburg, Germany) was used in cooperation with the Office of Student Affairs. EvaSys is a web-based software program for creating evaluation forms, carrying out evaluations online and/or in paper form as well as collecting and evaluating data.

At the beginning, general information on gender and the time required for preparation and follow-up for the course per week was asked (up to 30 min, 30-59 min, 60-120 min, more than 120 min). In the first set of questions, "General/Organization," it was evaluated whether the course took place regularly and on time, whether the guiding instructors had enough time for the training and whether the group size was exactly right for an effective learning process. This was followed by the second set of questions, "Structure/Clarity of the Subject Matter," and the third set of questions in the instructor-related part, "Commitment/Interactivity/Communication." In the fourth set of questions, "Media and Materials," it was asked whether the use of media contributed to a better understanding of the subject material and whether course materials (lecture notes) were provided. In the fifth set, the "Benefits" were evaluated. After that, questions were asked about the "Framework Conditions" relating to the premises and the technical equipment of the rooms. At the end of the questionnaire, the "Overall Assessment" was given with the items "I find the course to be very valuable for my education" and "How would you rate the course in the end?" (see table 2 (Tab. 2)).

The responses to the individual items could be given by marking an answer on a six-level modified Likert scale ("totally agree" to "totally disagree"), which followed the German school grade system of 1 (best) to 6 (worst). The evaluation reports contained the absolute and percentage frequency distributions of the determined items. The following descriptive statistics were used for each item: the mean average and the median as a measure of the central tendency and the standard deviation as the measure of dispersion.

Several items in a set of questions with the same sequence of answers (scale) were combined to a total value. This provided information on the total average and the total standard deviation of the set of questions.

In the present survey, only the paper questionnaire was used. All students of the three cohorts (T_1_, T_2_, T_3_) were surveyed, which makes it a complete survey. Information on the significance levels was not suitable, since the group of people surveyed were not a random sample. Instead, the descriptive statistics of the population were determined.

## Results

96% (69 of 72) of the students participated in the first evaluation in 2012/2013 (T_1_); of these 68% were women. 60.3% of the students reported requiring up to 30 min per week for preparation and follow-up for the course. 33.8% reported requiring 30-59 min, and 5.8% of the participants took more than 60 min. 

In the following year, 2013/2014 (T_2_), 92% (45 of 48 students) participated; of these 62% were women. 86.5% of the students reported requiring up to 30 min per week for preparation and follow-up for the course. 13.5% reported requiring 30-59 min. The 2014/2015 evaluation (T_3_) included 94% (45 of 48 students); of these 67% were women. 67.4% of the students reported requiring up to 30 min per week for preparation and follow-up for the course. 32.6% reported requiring 30-59 min. 

**Figure 1:**
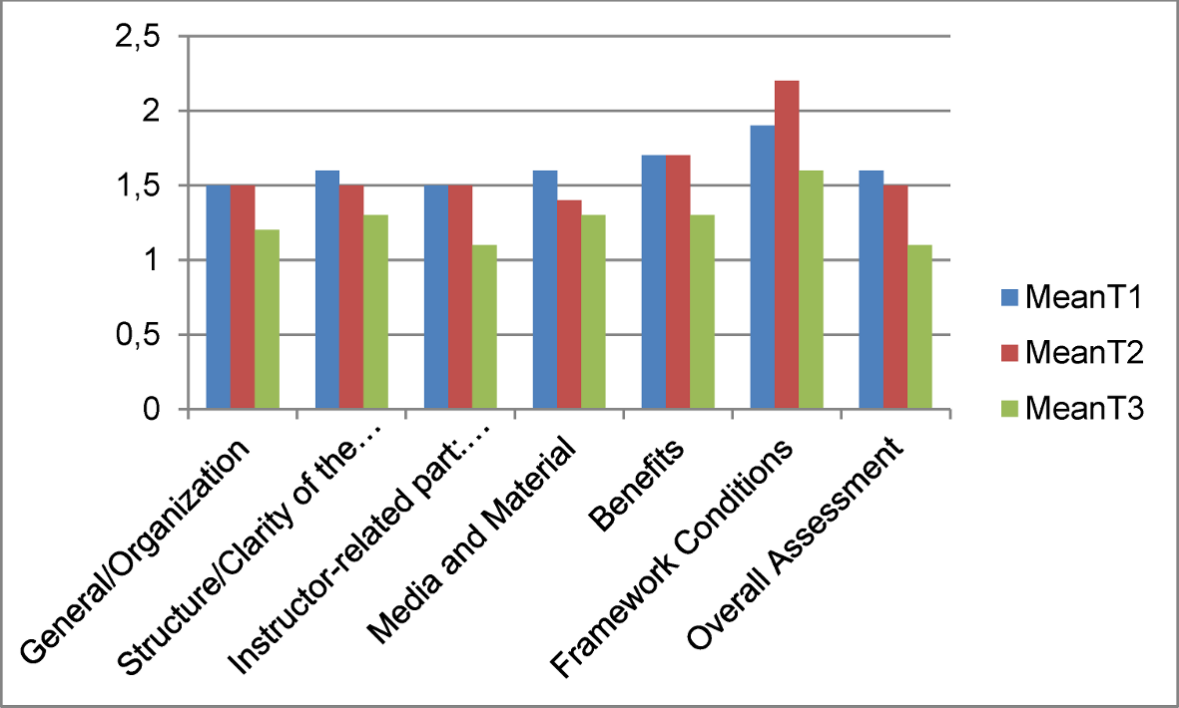
Total average value in relation to the individual sets of questions from table 2

Figure 1 (Fig. 1) shows the results in relation to the sets of questions. On the whole, a higher grading of the individual items could be observed from T_1_ to T_3_. At T_1_ and T_2_ "General/Organization" was given a mean (M) a mean of M=1.5 (SD=0.7) and at T_3_ a mean M=1.2 (SD=0.3). The item "Overall Assessment" yielded M_T1_=1.6 (SD=0.6), M_T2_=1.5 (SD=0.5) and M_T3_=1.1(SD=0.3). 

The items for "Structure/Clarity of the Subject Matter" are listed in table 3 (Tab. 3). At point T_1_ the item "Research results were included in the course" was still evaluated with M=2.7 (SD=1.1). At T_2_ a mean was M=1.5 (SD=0.8) and at T_3_ M=1.8 (SD=1.1). Table 4 (Tab. 4) lists the items of the instructor-related part. For all items, a considerable increase in the student evaluation could be seen at the time of T_3_. In T_1_ and T_2_ the item "The instructor actively included students in the course" was still given mean value of 2.1 (SD=0.9) and at T_3_ a mean of 1.2 (SD=0.5). 

## Discussion

In the conception and development of the Clinical Tooth Shade Differentiation Course, clinical methods for determining tooth shade were integrated in the preclinical propedeutics to prepare the students for the clinical stage of their studies. The theoretical and practical skills for determining tooth shade were continuously adapted to the clinical treatment situation during the course presented here. Starting with shade training with the TT and the TTB, students first practiced the manual shade-taking procedure on a phantom patient and then on one another in role playing. The digital shade-taking procedure first took place on a test specimen, then on a phantom patient and subsequently in role playing. This resulted in a learning spiral with increasingly difficult sequences of practical skills in visual and digital tooth shade-taking. 

The three cohorts varied in the number of eligible students due to different numbers of new admissions. The evaluation of the students showed a positive grading of the course. Only a few students did not participate in the survey. The curriculum was supervised by an instructor over the indicated period of time. Nevertheless, minor differences appeared in the response behavior of the three cohorts. An improvement in the evaluation can be seen in almost all items at the time of T_3_. One reason for this could be a more intensive student/instructor interaction in the individual classes (lectures, seminars). The course was also adapted and optimized by the instructor over the specified period of time. The interests of the learners were taken into account via the evaluations. 

Lectures and seminars in the presented course were structured according to didactic aspects to maintain the attention and motivation of the participants. The sandwich principle was used to implement activating forms of teaching in day-to-day teaching [13], [14]. Learning processes can be promoted by alternating activity and passivity as well as individual and collective learning phases [13], [14]. It is also possible to maintain the concentration of listeners by changing the method in a lecture or seminar [15], [16]. The practical exercises for visual and digital shade-taking procedures took place in a small group of up to six students. The joint exercise for clinical shade-taking was carried out in groups of two in role playing under clinical conditions. The meta-analysis of Hattie showed that learning processes that are integrated in social situations such as group or partner work are more effective [18]. The practical skills for manual and digital shade-taking were taught according to the Peyton method [17]. In a study by Heni et al., the Peyton method was assessed to be a very effective teaching method for imparting practical skills in a skills lab [19]. The study by Krautter et al. also suggests this [20].

Students have their own view and perspective on teaching and learning in the educational process. Evaluations give students the possibility of having a certain amount of say in the teaching process. This student feedback allows instructors to refine their own approaches [21], [22], [23] and to make adjustments if necessary to create a better learning environment. The overwhelming majority of published articles in the area of dental education use student evaluations to assess innovations in teaching [24], [25], [26], [27], [28], [29], [30], [31]. The investigation of Subramanian et al. [32] considers students as collaborative partners in the educational process.

To maintain the quality of the presented project over the long term, it is important to continue to evaluate the teaching situation. Further prospective studies with objective measuring methods are necessary for a final assessment. The goal should be to teach interdisciplinary material progressively in the preclinical and clinical stage of studies, which in turn should result in better and more competent care of patients. The use of electronic aids such as e-learning can additionally support this process in dental medicine [33], [34], [35], [36], [37], [38]. 

## Conclusions

The Clinical Tooth Shade Differentiation Course has been regularly offered as an obligatory part of phantom course II since Winter Semester 2012/13. The course presented here conceptually shows how practical dental skills can be taught in relation to a theoretical and clinical context. In view of the national competence-based catalog of educational objectives, educational objectives were taken from the role of a dental expert and can be supplemented further. The objectives can be transferred to other dental faculties.

## Acknowledgements

The authors would like to thank Mr. Henze for analyzing the evaluation forms. We would also like to thank Vita Zahnfabrik for supplying the TTBs.

## Competing interests

The authors declare that they have no competing interests. 

## Figures and Tables

**Table 1:**
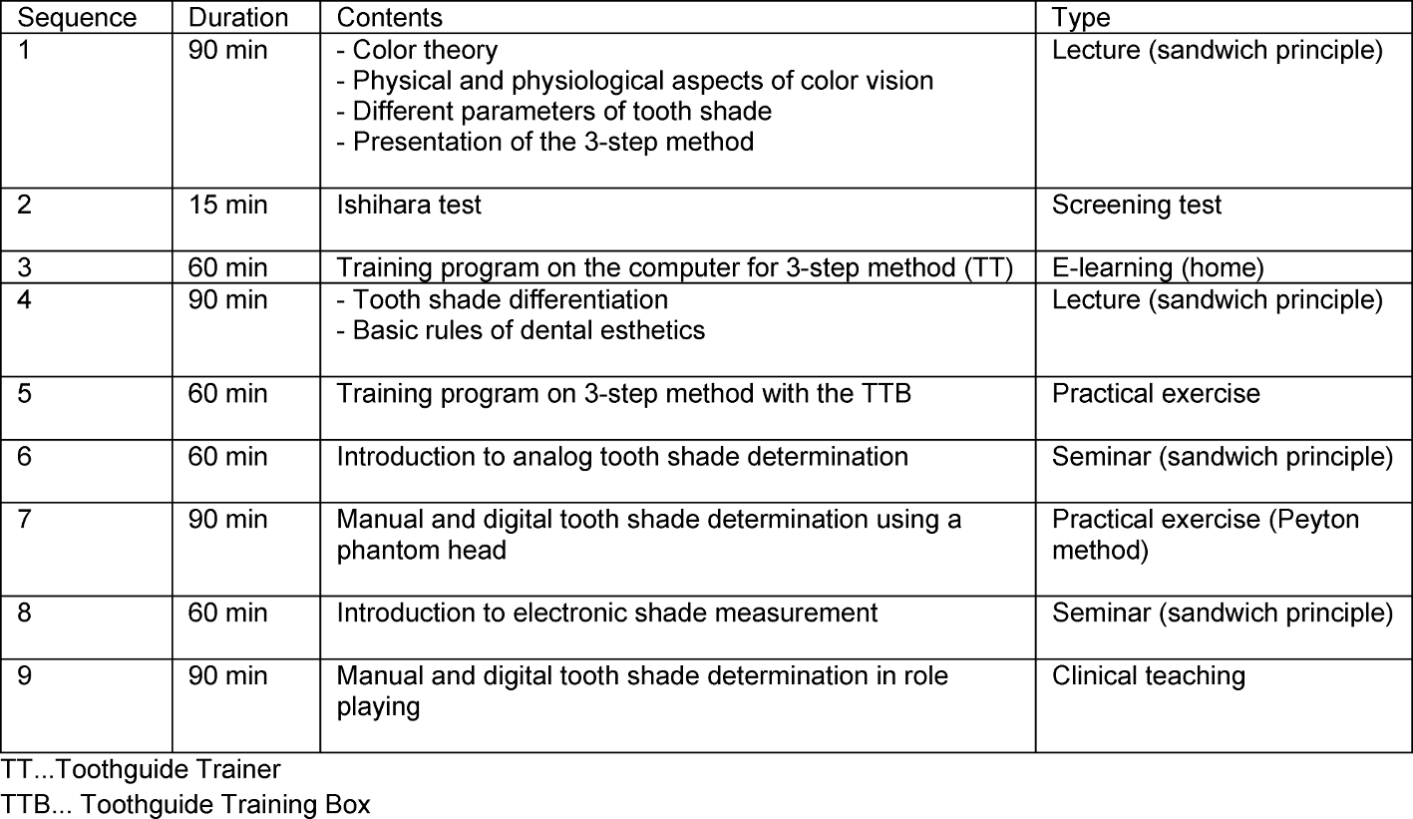
Methodical-didactic implementation of the Clinical Tooth Shade Differentiation Course

**Table 2:**
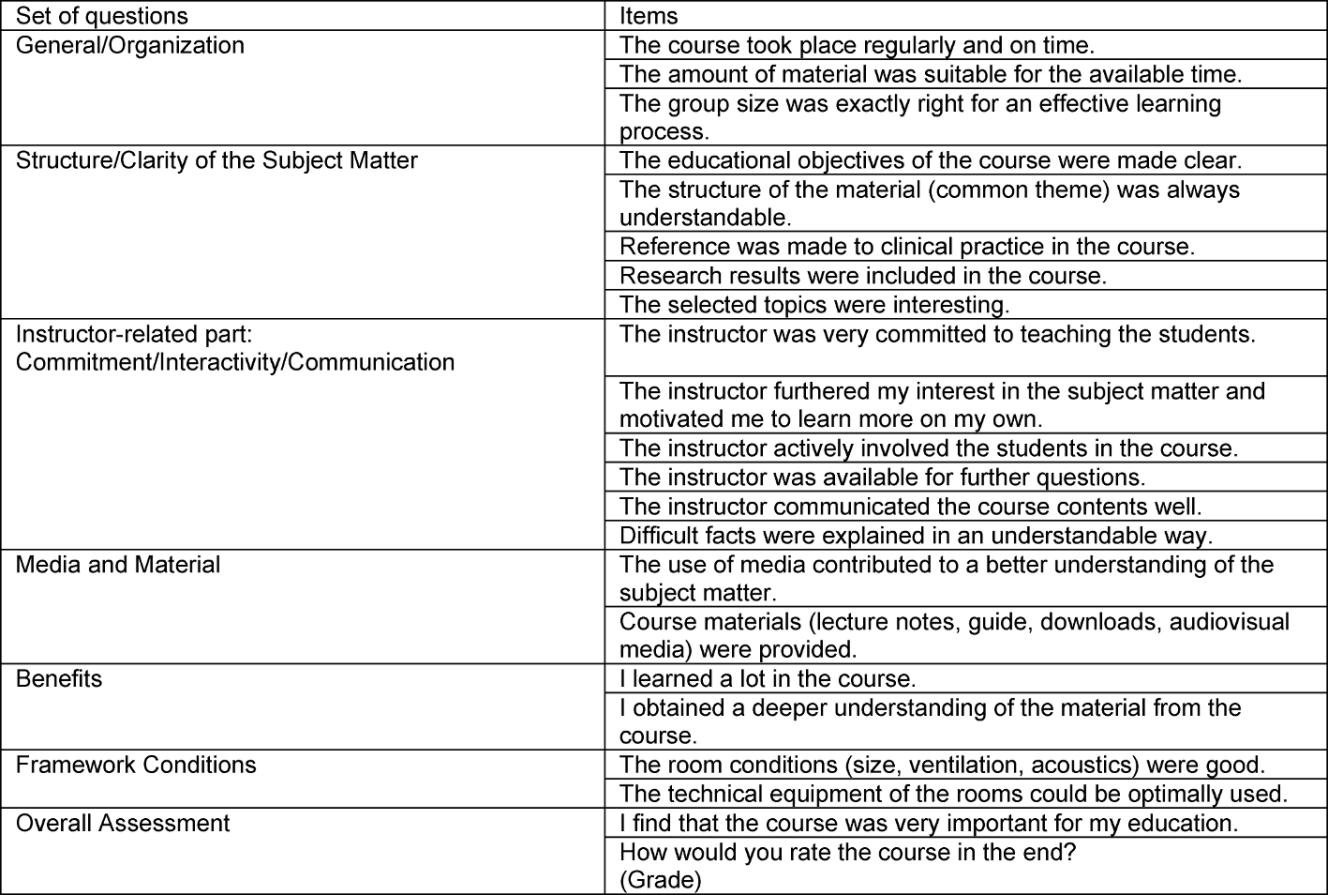
Set of questions with the individual items of the student evaluation

**Table 3:**
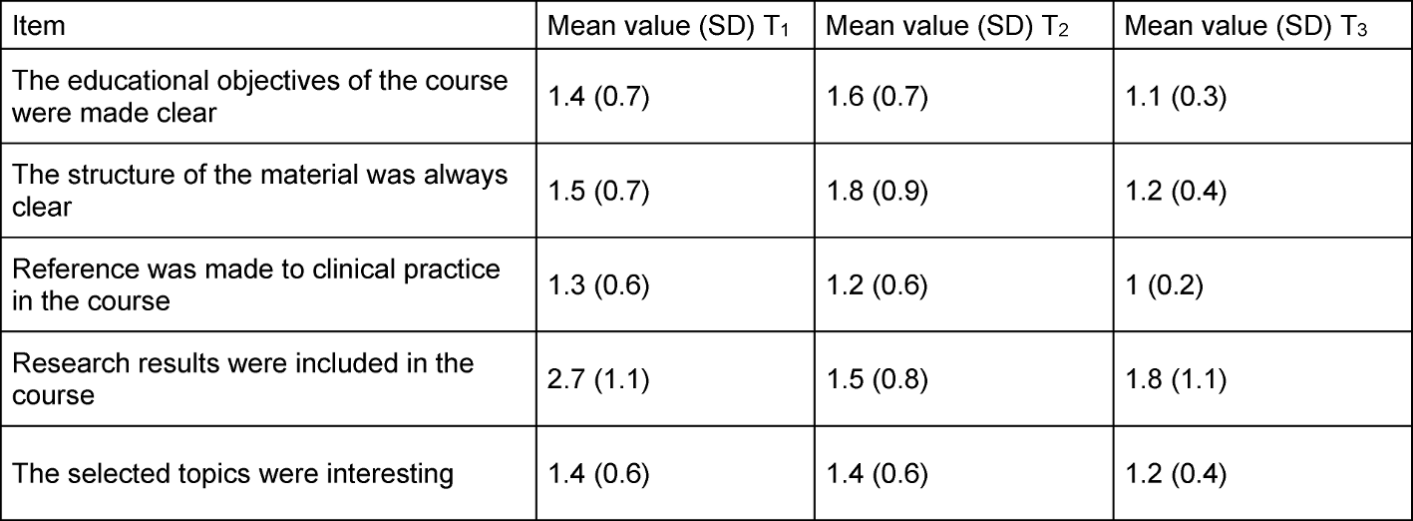
Mean value and standard deviation of the set of questions in Structure/Clarity of the Subject Matter

**Table 4:**
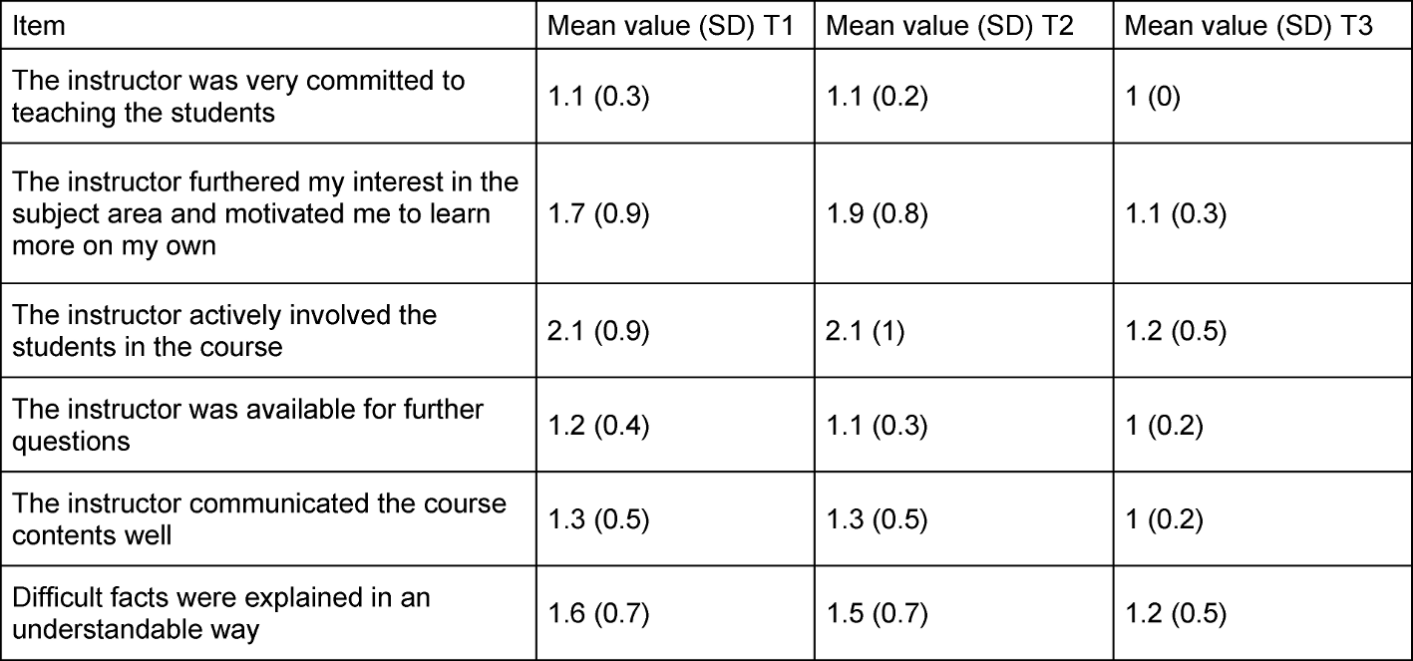
Mean value and standard deviation of the items in the set of questions in the instructor-related part
